# A Highly Compact Antipodal Vivaldi Antenna Array for 5G Millimeter Wave Applications

**DOI:** 10.3390/s21072360

**Published:** 2021-03-29

**Authors:** Amruta Sarvajeet Dixit, Sumit Kumar, Shabana Urooj, Areej Malibari

**Affiliations:** 1 Symbiosis Institute of Technology, Symbiosis International Deemed University, Pune 412115, India; amrutaamalode@gmail.com; 2 Department of Electrical Engineering, College of Engineering, Princess Nourah bint Abdulrahman University, Riyadh 84428, Saudi Arabia; smurooj@pnu.edu.sa; 3 College of Engineering, Princess Nourah bint Abdulrahman University, Riyadh 84428, Saudi Arabia; aamalibari@pnu.edu.sa; 4 Department of Computer Science, Faculty of Computing and IT, King Abdulaziz University, Jeddah 80200, Saudi Arabia

**Keywords:** antipodal Vivaldi antenna (AVA), millimeter wave, compact, 5G applications, corrugations

## Abstract

This paper presents a compact 1 × 4 antipodal Vivaldi antenna (AVA) array for 5G millimeter-wave applications. The designed antenna operates over 24.19 GHz–29.15 GHz and 30.28 GHz–40.47 GHz frequency ranges. The proposed antenna provides a high gain of 8 dBi to 13.2 dBi and the highest gain is obtained at 40.3 GHz. The proposed antenna operates on frequency range-2 (FR2) and covers n257, n258, n260, and n261 frequency bands of 5G communication. The corrugations and RT/Duroid 5880 substrate are used to reduce the antenna size to 24 mm × 28.8 mm × 0.254 mm, which makes the antenna highly compact. Furthermore, the corrugations play an important role in the front-to-back ratio improvement, which further enhances the gain of the antenna. The corporate feeding is optimized meticulously to obtain an enhanced bandwidth and narrow beamwidth. The radiation pattern does not vary over the desired operating frequency range. In addition, the experimental results of the fabricated antenna coincide with the simulated results. The presented antenna design shows a substantial improvement in size, gain, and bandwidth when compared to what has been reported for an AVA with nearly the same size, which makes the proposed antenna one of the best candidates for application in devices that operate in the millimeter frequency range.

## 1. Introduction

Millimeter-wave applications are used in medical imaging, the military, satellites, and 5G communication. The current 4G communication for mobile phones provides a moderate data rate and capacity and suffers from spectrum shortage [1]. The evolutionary 5G technology is the panacea of current mobile communication issues, and it can be used to provide various revolutionary services. The international telecommunication union (ITU) has specified various specifications of 5G technology; for example, spectrum efficiency should be up to 9 bits/s/Hz, there should be a high data rate from 2 Gbps to 20 Gbps, connection density should be 1 million/km ${}^{2}$, mobility should be up to 500 km/h, and operating frequency should be below 6 GHz (3 GHz to 5.5 GHz) and above 6 GHz (28 GHz, 38 GHz, and 70 GHz) [2,3]. The main requirement for the deployment of 5G technology is that devices should work in the millimeter-wave frequency range [2], but working in this range can cause devices to suffer from higher path losses [4]. Such path losses at higher frequencies can be reduced by using the high-gain antenna array. This fact provoked the design of a compact antenna that can work at such a high-frequency band with enhanced gain and a stable radiation pattern. The antipodal Vivaldi antenna (AVA), which was invented by Gazit [5], is one of the best candidates as a 5G antenna that works in the millimeter-wave range. The Vivaldi antenna, which was invented by Gibson [6], operates at high frequencies and provides a wide bandwidth. the AVA is preferred over the Vivaldi antenna because it provides a wider bandwidth, high gain, a nearly-constant radiation pattern, and it is easy for fabrication. A single AVA cannot meet all the requirements of a 5G antenna, such as high gain (above 8 dBi), efficiency (above 85%), a wide bandwidth (above 6 GHz), and stable radiation patterns; thus, it is necessary to design a highly compact AVA array that fulfils the above-mentioned requirements [7,8].

Various antenna performance enhancement techniques have been investigated by researchers [3]. The gain of the AVA can be increased by using an array [9,10,11]. In [12], a 1 × 4 AVA array with substrate integrated waveguide (SIW) and corrugations was implemented to obtain a high gain of 23 dBi. Furthermore, the bandwidth of the AVA can be increased by using slots in AVA flare [13,14]. In [15], a wideband antenna was designed to operate from 3.4 GHz to 40 GHz by incorporating slots in the AVA. The finalization of the dimension and position of the slots is a challenging task [16]. Additionally, the AVA can be made compact by incorporating corrugations [17,18]. The corrugations are of a triangular, sinusoidal, rectangular, or square shape. Out of these shapes, the rectangular shape is frequently used in the literature. Some researchers have implemented the AVA with metamaterial [10], dielectric lens [19], and balanced AVA [20] to improve the gain. The balanced AVA consists of three layers: the top and bottom layers function as conductors, and the middle layer functions as a ground. This structure provides equal electric field distribution on both sides to the ground, but the antenna design is complex [21]. Similarly, the design and placement of the metamaterial unit cells are also complex. Furthermore, after incorporating a dielectric lens, the size of the antenna increases [22]. Moreover, some researchers have implemented the AVA with parasitic patches to improve the gain, but this enhancement is not substantial, and the parasitic patch adversely affects the size of the antenna [23]. Hence, it is a challenging task to design a compact antenna with the required performance parameters.

This paper presents a 1 × 4 AVA array with corrugations to achieve a high gain, wide bandwidth, and size reduction. Due to the non-ideal directional characteristics of the practical antennas, the energy radiated from each array antenna element is received by other array elements. The partial amount of this energy may be re-scattered in a different direction, which is known as mutual coupling [24]. If the distance among the array elements is minimal, then the interchange of energy (energy scattering) increases, thereby increasing mutual coupling. Furthermore, this re-scattered energy radiates in any direction other than the intended one, which gives rise to increases in side-lobe levels (SLL). Hence, because of the basic structure of the antenna array, there are more side-lobe levels (SLLs) and mutual coupling of array elements [4]. More SLLs affect the radiation pattern, while mutual coupling alleviates the antenna bandwidth. Both of these issues are mitigated by incorporating corrugations in AVA flares. The corrugations reduce back- and side-lobe levels, and they also improve the bandwidth [25]. Out of the various performance enhancement methods, corrugation is easy to implement, and it is a very effective technique. Furthermore, in [26], comb-shaped (rectangular) corrugations are etched on the AVA flare to identify voids in concrete beams. In [27], rectangular corrugations with a constant, increasing, and decreasing size are demonstrated. In [27], it is proved that AVA performance can be effectively improved by using corrugations with either a constant or decreasing size. In this paper, the constant size corrugation is incorporated in the AVA.

The array elements can be fed by using either series or corporate feeding. In series feeding, different antenna elements are excited differently to alleviate the SLLs. Even though the array with a series feed is compact, it provides a narrow bandwidth, which is not desirable [28]. In corporate feeding, different antenna elements are excited equally, and it enhances the bandwidth [29]. This paper presents the design of a 1 × 4 AVA array with corporate feeding to achieve a wide bandwidth for 5G applications that operate in the millimeter-wave frequency range. The detailed design and parametric study of the AVA array with corrugations are given in Section 2. Section 3 proves the importance of the proposed antenna with the help of various simulated and measured results. Finally, the overall findings of this paper are summarized in the Conclusion Section 4.

## 2. Design of Proposed AVA Array

### 2.1. Single AVA Design

**Figure 1:**
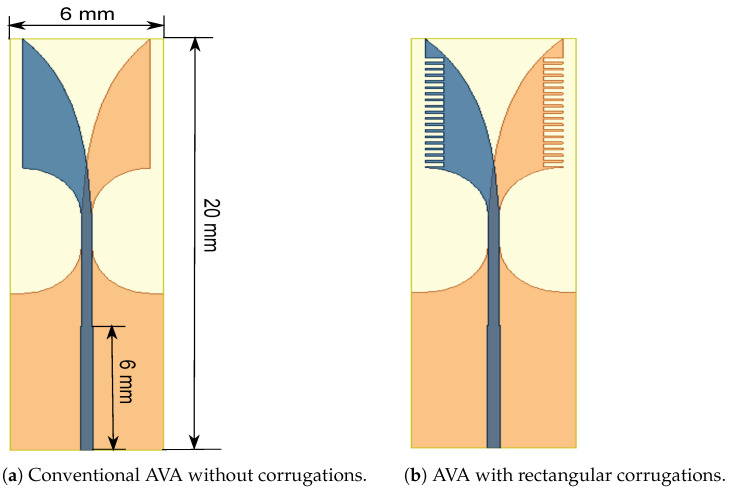
Design of the proposed single-patch antenna.

The single-patch conventional AVA and the proposed AVA with corrugation are shown in Figure 1. They are fabricated on RT/duroid 5880 substrate, which has 2.2 dielectric permittivity and a loss tangent of 0.001. The antenna was designed using a high-frequency structure simulator (HFSS) version 2020 R2. Its length is 20 mm, its width is 6 mm, and its substrate thickness is 0.254 mm. The proposed AVA has a top patch that acts as a radiator and a bottom patch that acts as a ground. Both the ground and radiator patches are mirror images of each other. These patches are formed by using an inner circular arc and an outer exponential curve whose equation is given below [30].

(1) $$Y_{1}=\pm \left(C1e^{350x}-C2\right)\;\left(8.65\leq x\leq 17.25\right)$$

where C1 = 0.00678 mm and C2 = 3.4 mm

**Figure 2:**
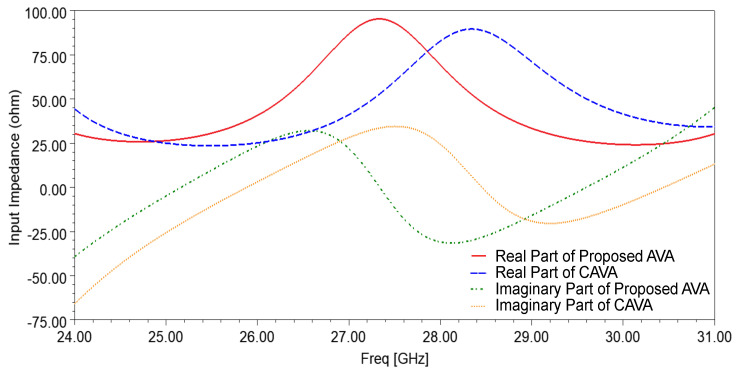
Input impedance of the single-patch AVA with and without corrugations.

**Figure 3:**
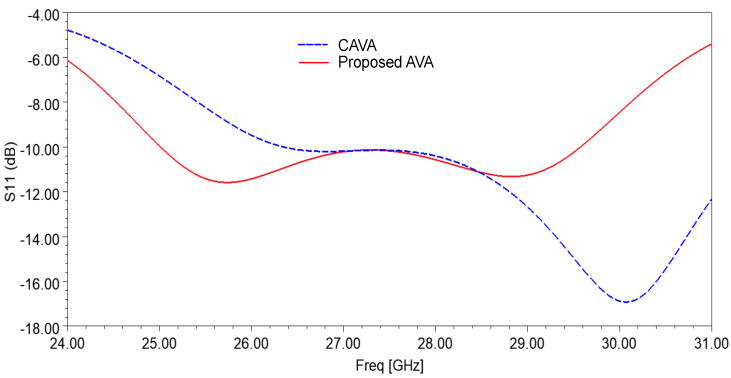
Reflection coefficient of the single-patch AVA with and without corrugations.

The input impedance plot of the single-patch AVA with and without corrugations is shown in Figure 2. The slots of corrugations introduce the RLC circuit, which results in a change in impedance and resonance frequency of the antenna. As shown in Figure 2, the input impedance plot is shifted to the lower frequency range after incorporating corrugations in the AVA. This shifting in input impedance results in a decrease in lower cut-off frequency, as shown in Figure 3. The lower cut-off frequency of the conventional AVA is 26.33 GHz, whereas the lower cut-off frequency of the proposed AVA with corrugations is 25 GHz. Hence, corrugations result in a change in the frequency response of the antenna. By optimizing the dimensions of corrugations, bandwidth and input impedance enhancement can be achieved. The optimized corrugations depth is 0.7 mm, the width of the corrugation teeth is 1mm, and the width of the corrugation slot is 2 mm.

**Figure 4:**
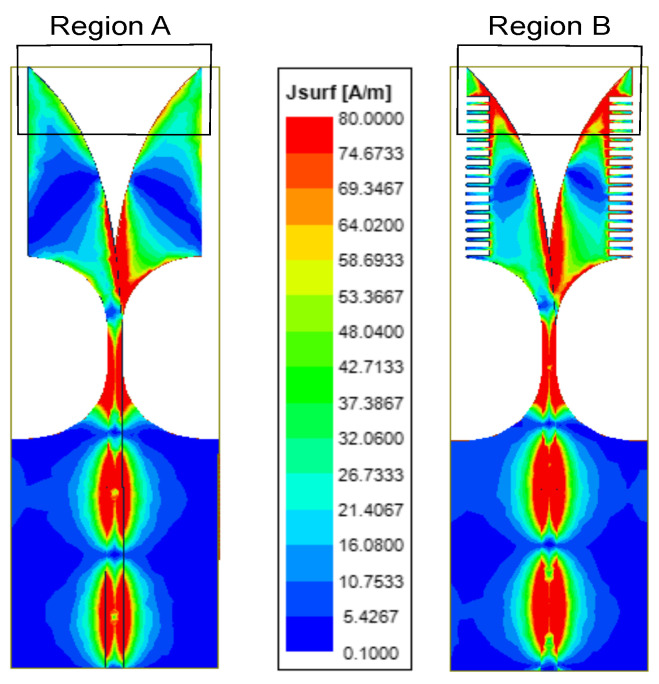
Electric field distribution of the single-patch AVA with and without corrugations at 27.5 GHz.

**Figure 5:**
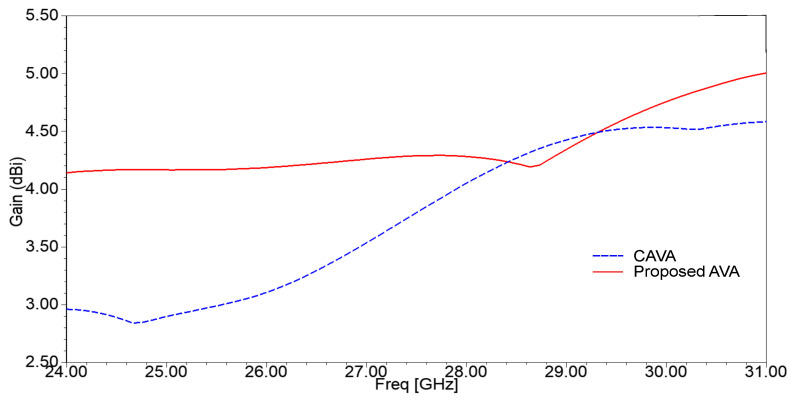
Gain versus frequency plot of the single-patch AVA with and without corrugations.

Next, the vital role of corrugations in electric field enhancement is proved in Figure 4. In this figure, region A shows that the concentration of the electric field is less than that of the electric field concentration of region B. Thus, corrugations also contribute to enhancing radiation, resulting in a substantial gain improvement, as shown in Figure 5. The range of gain for the AVA without corrugation is 3.2 dBi to 4.45 dBi, whereas the range of gain for the AVA with corrugations is 4.2 dBi to 4.5 dBi, which is almost constant. Thus, corrugations improve the gain of an antenna.

### 2.2. 1 × 4 AVA Array Design

**Figure 6:**
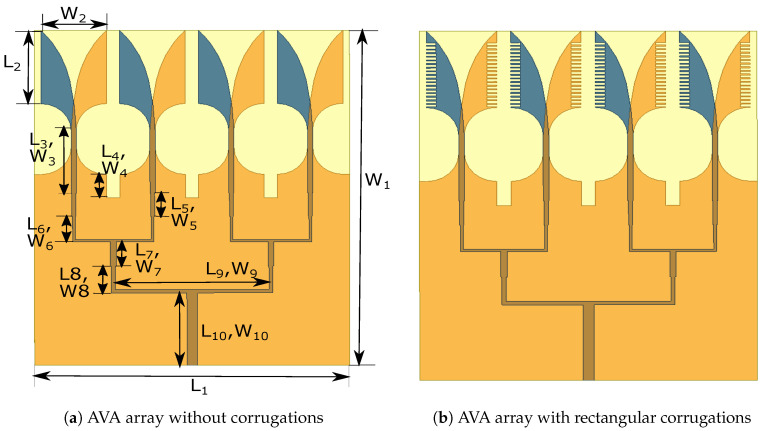
Design of proposed antenna.

**Figure 7:**
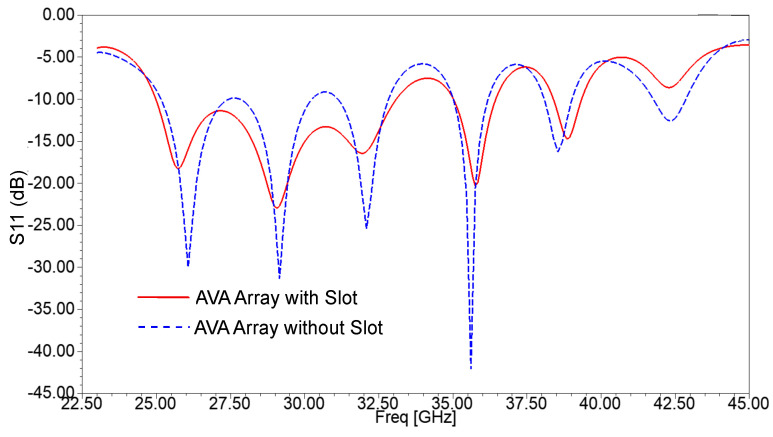
Reflection coefficient of the AVA array with and without slots in the ground.

The 1 × 4 AVA array was formed by using the AVA with the corrugations shown in Figure 6a,b. The length of the proposed AVA is 28.8 mm and its width is 24 mm. The rectangular slots are incorporated in the ground plane to improve the frequency response of the AVA array depicted in Figure 7. The dotted graph is S11 of the AVA array without slots and corrugations, whereas the continuous graph is S11 of the AVA array with slots in the ground plane and without corrugations. This figure shows an improvement in the frequency response of the AVA, particularly in the frequency range of 25 GHz to 33 GHz. The slot structure changes the RLC circuit, which results in a change in the frequency response of the antenna.

**Figure 8:**
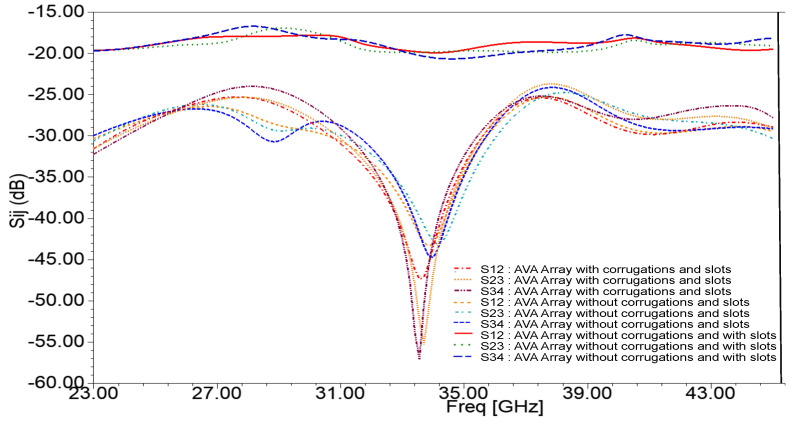
Simulated mutual coupling between array elements after removal of 1 × 4 power divider.

**Figure 9:**
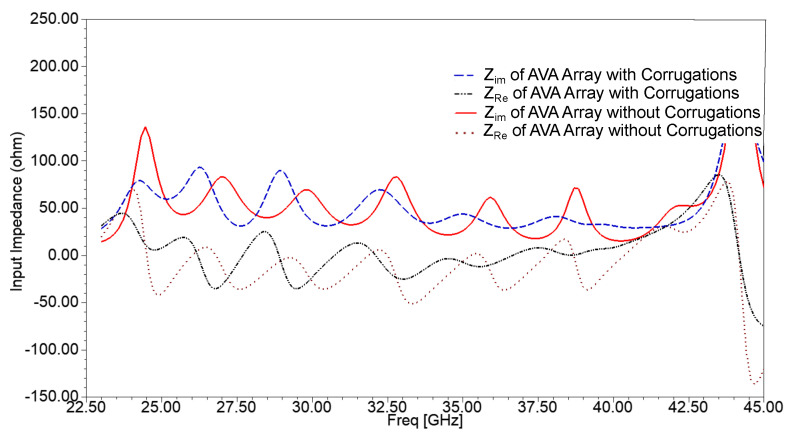
Input impedance of the AVA array with and without corrugations.

The mutual coupling between array elements is shown in Figure 8. As depicted in Figure 8, the mutual coupling of the 1 × 4 AVA array is in the acceptable range. The mutual coupling improved after the introduction of slots in the ground plane, and it was further enhanced by incorporating corrugations in the AVA array. The slots in the ground plane provided isolation enhancement of 24 dB at 34 GHz, while corrugations improved the isolation by 36.4 dB at 33.56 GHz. Figure 9 indicates that the input impedance of the AVA array varied from 27 Ω to 100 Ω, whereas the input impedance of the AVA array with corrugations changed from 40 Ω to 63 Ω over the frequency range of 24.2 GHz to 40.5 GHz. Thus, the corrugations also contributed to improving input impedance matching.

**Figure 10:**
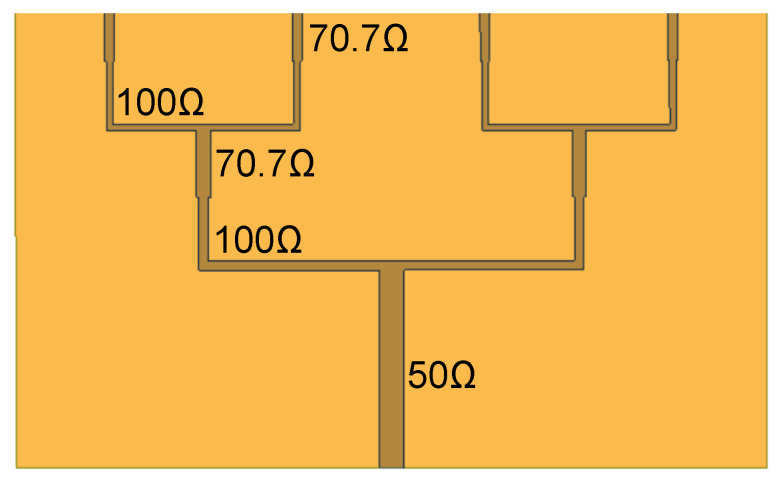
Feeding network of the AVA array

Input impedance was also enhanced by using a corporate feeding network. The corporate feeding is present at the top to feed the top patches with equal power. The impedance of each branch of the feeding network is given in Figure 10. Initially, a 50 Ω feeding line was used, and then it was bifurcated into two 100 Ω feeding lines. This structure of the two 100 Ω feeding lines and the single 50 Ω feeding line resembles a ‘T’ junction. This ‘T’ junction was repeated to obtain four feeding lines for the four top patches. Additionally, the λ/4 transformer was used between the 50 Ω and 100 Ω feeding line for good impedance matching. The feeding network provided nearly 50 ± 10 Ω impedance, and it was optimized using HFSS.

**Figure 11:**
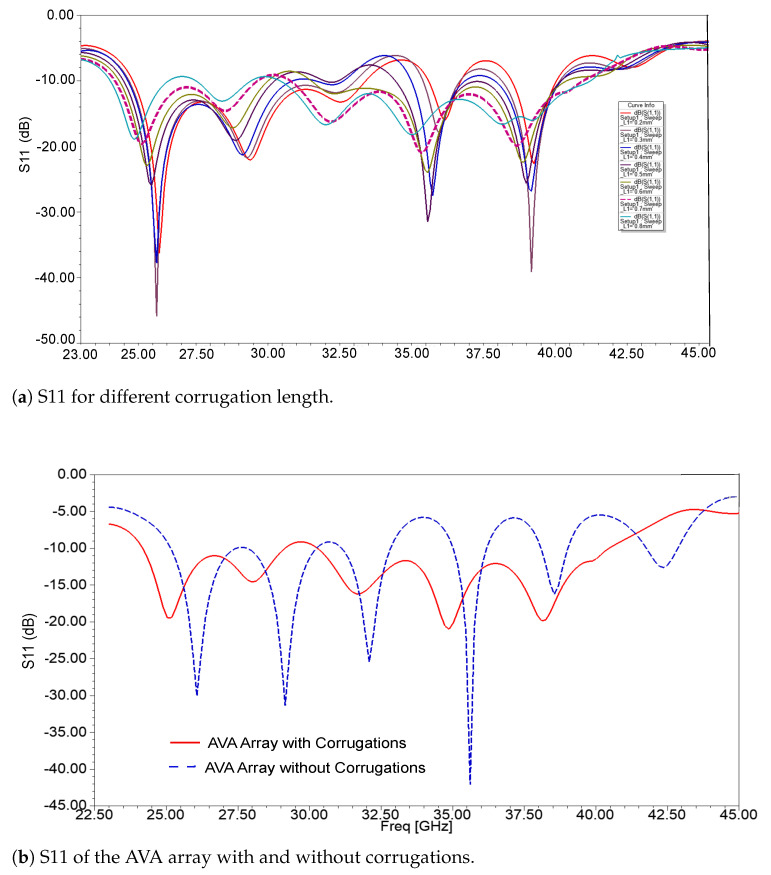
Reflection coefficient of the designed antenna.

**Figure 12:**
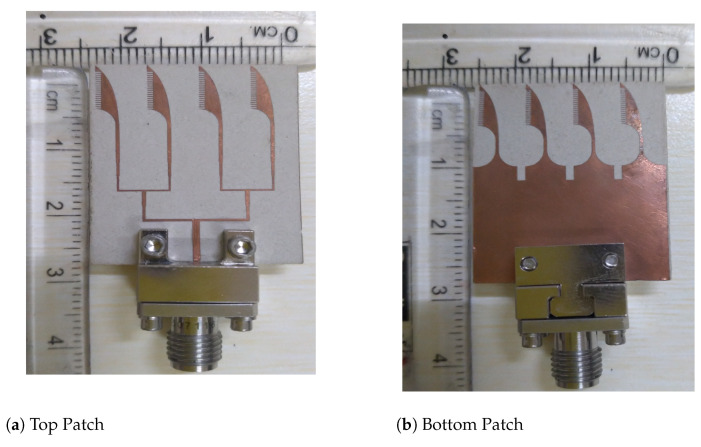
Fabricated proposed antenna.

Corrugations were also introduced in both the top and bottom patches. The structure of corrugation resembles a comb shape, which means that its structure is rectangular, and the size of the rectangle is uniform over the complete top and bottom patches. The corrugations change the capacitance, inductance, and resistance of an antenna, which positively reflects on bandwidth enhancement. The dimension of corrugation’s length slot was optimized using HFSS, as shown in Figure 11a. This figure demonstrates that the best reflection coefficient result was obtained for a corrugation length of 0.7 mm. The width of the slot and teeth of the corrugations are 2 mm and 1 mm, respectively. Furthermore, the vital role of corrugations in the bandwidth improvement is shown in Figure 11b. This figure displays the reflection coefficient plots of the AVA array with and without corrugations. It shows that there are tri-bands in the AVA array, which are 25.09 GHz–32.9 GHz, 34.97 GHz–36.24 GHz, and 38.03 GHz–39.07 GHz, whereas a dual-band from 24.19 GHz–29.15 GHz and 30.28 GHz–40.47 GHz was achieved after incorporation of corrugations in the AVA array. This wide frequency band contains three important frequency bands of 5G, communications which are 24.25 GHz–29.5 GHz, 31.8 GHz–33.4 GHz, and 37.5 GHz–40.5 GHz. The fabricated antenna is shown in Figure 12, and the optimized dimensions are given in Table 1.

## 3. Results and Discussion

**Figure 13:**
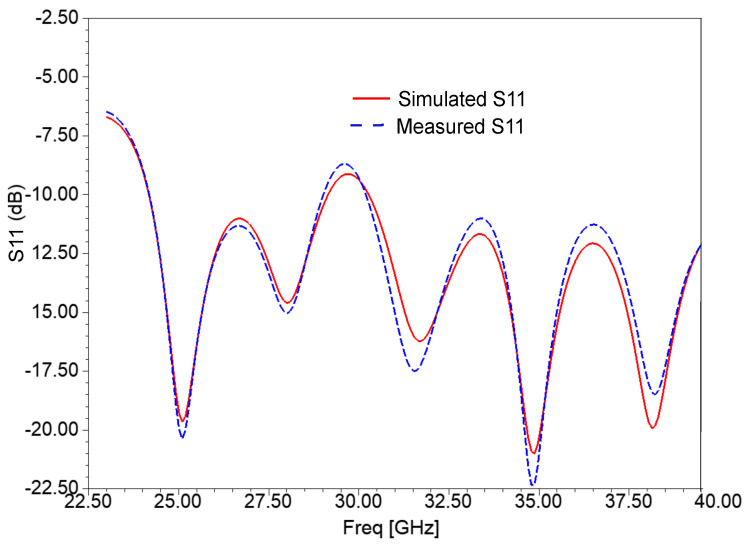
Simulated and measured S11 of the AVA array with corrugations.

The fabricated antenna was tested using an N5224A performance network analyzer (PNA). The simulated and measured reflection coefficient (S11) results are shown in Figure 13. Because of unavoidable fabrication drawbacks, there were minor dissimilarities between the measured and simulated results. The simulated frequency range is from 24.19 GHz–29.15 GHz to 30.28 GHz–40.5 GHz, whereas the measured operating frequency range is from 24.19 GHz–29.02 GHz to 30.20 GHz–40.33 GHz. The frequency ranges of both simulated and measured results where S11 is above −10 dB are not allotted for 5G applications. Hence, the designed antenna covers 24.25 GHz–29.5 GHz, 31.8 GHz–33.4 GHz, and 37.5 GHz–40.5 GHz frequency bands of 5G communications.

**Figure 14:**
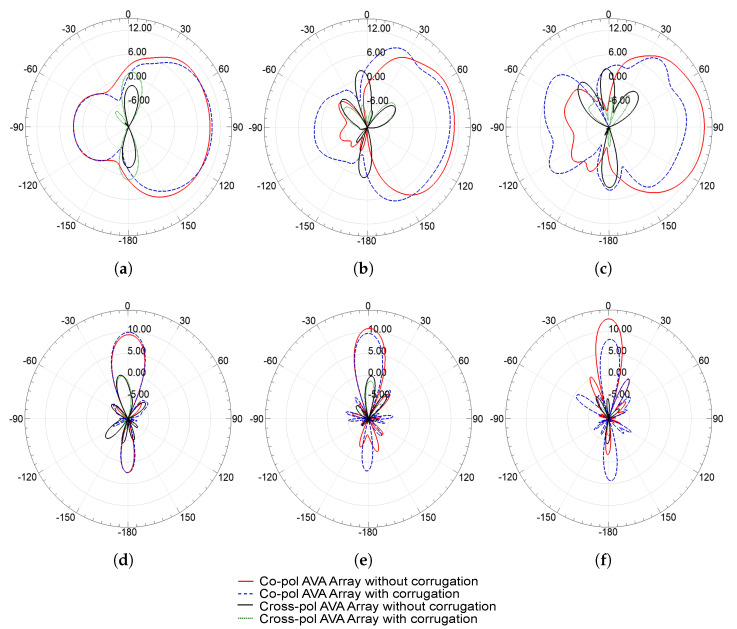
Simulated co-polarization and cross-polarization of E plane (**a**–**c**) and H plane (**d**–**f**).

The co- and cross-polarization of elevation (H-plane) and azimuth (E-plane) planes at 25.5 GHz, 31.5 GHz, and 38 GHz are shown in Figure 14. This figure proves that the radiation patterns at different frequencies are almost the same, and, thus, the antenna performance is frequency independent. Therefore, the designed antenna provides a stable radiation pattern. In both the E and H planes, the front lobe of the AVA array with corrugations is higher than the front lobe of the AVA array without corrugations at various frequencies. Furthermore, the back- and side-lobe levels are also alleviated in the AVA array with corrugations as compared to the AVA array without corrugations. Hence, this figure depicts the enhancement in the front-to-back ratio and gains by employing corrugations in the AVA flares. Next, as the element spacing is 5 mm, which is less than the center wavelength, grating lobes are absent in all radiation patterns. Importantly, the cross-polarization of the AVA array with corrugations is lower than the cross-polarization of the AVA array without corrugations.

**Figure 15:**
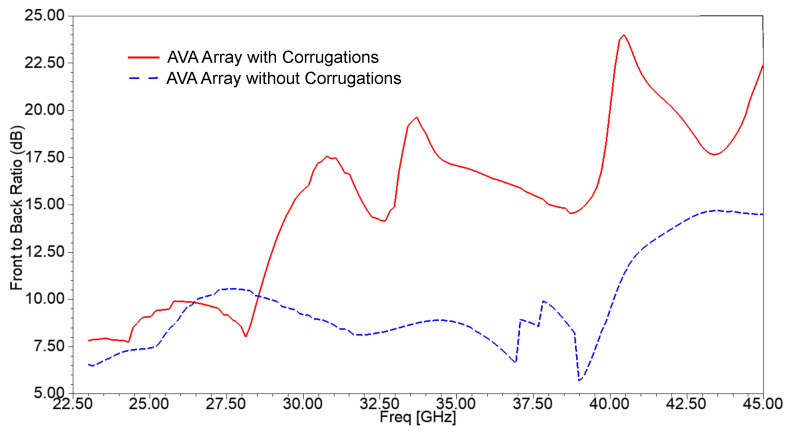
Simulated front-to-back ratio of the AVA array with and without corrugations.

The importance of corrugations for the improvement of the front-to-back ratio (FBR) can be clearly observed in Figure 15. The FBR of the AVA without corrugations is in the range of 5.7 dB to 10.5 dB, whereas the FBR of the AVA with corrugations is from 8 dB to 24 dB. The highest FBR of the AVA with corrugation is 24 dB at 40.45 GHz, whereas the highest FBR of the AVA without corrugations is 10.5 dB at 27.54 GHz. As a consequence of this, it is proved that the corrugation increases the front lobe and reduces the back lobe, which results in the enhancement of FBR and gain.

**Figure 16:**
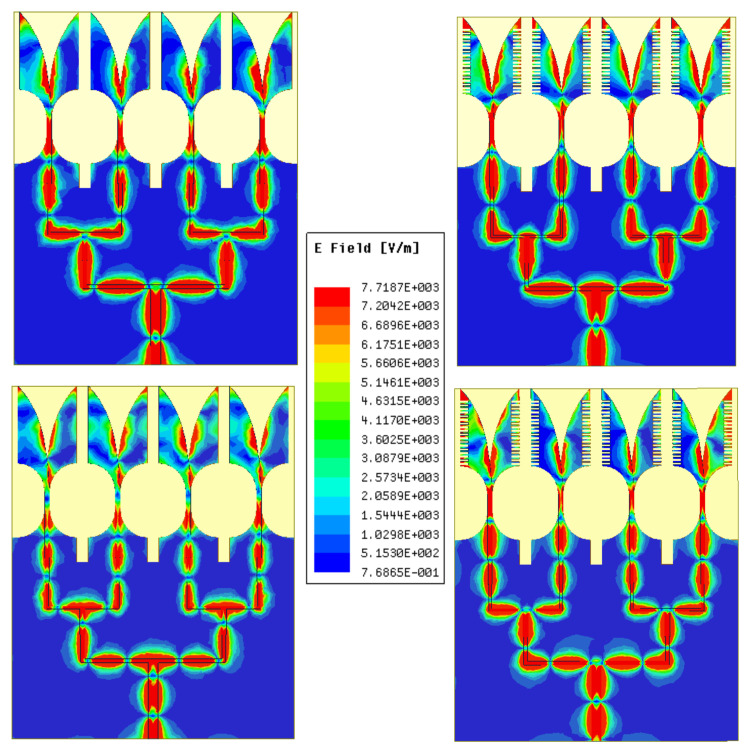
Electric field distribution of the AVA array with and without corrugations at 25 GHz (**upper**) and 34 GHz (**lower**).

Figure 16 shows the simulated electric field distribution of the AVA array with and without corrugations at 25 GHz (upper) and 34 GHz (lower). It shows that the power divider networks are optimized correctly to distribute the input power equally to all four antenna elements. The AVA array structure provided plane-like waves. In a 1 × 4 AVA array with corrugations, the concentration of the electric field is higher at the edges as compared to the AVA array without corrugations. Furthermore, the AVA array with corrugation enhanced directivity as compared to the AVA array without corrugations, which resulted in gain enhancement.

**Figure 17:**
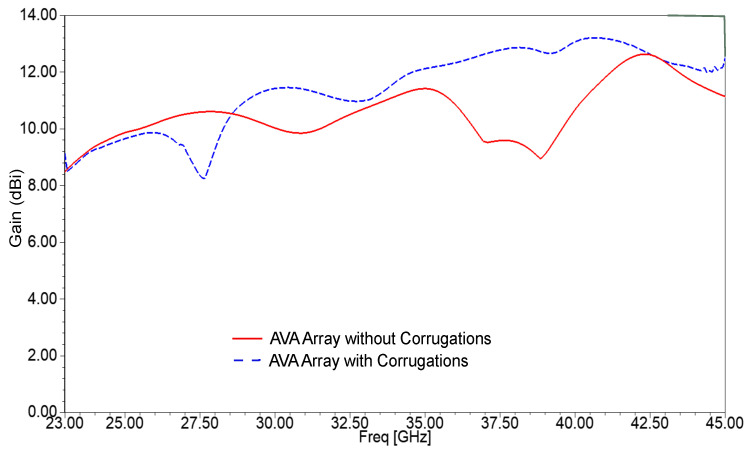
Simulated gain plots of the AVA array with and without corrugations.

**Figure 18:**
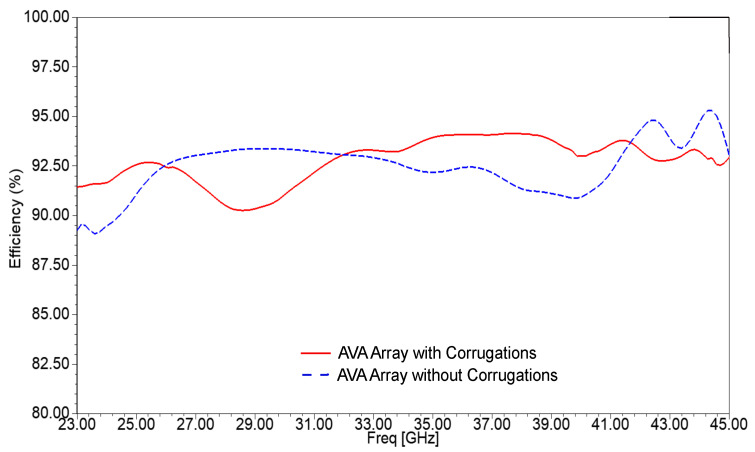
Simulated efficiency of the AVA array with and without corrugations.

The contribution of corrugation to gain enhancement is also shown in Figure 17. The gain of the AVA array without corrugations is from 8.47 dBi to 12.63 dBi, whereas the gain of the AVA array with corrugations is from 8 dBi to 13.2 dBi. Thus, the peak gain was enhanced by 0.57 dBi, and the gain variation was reduced to some extent. The gain of the AVA with corrugations is low at 27.5 GHz, which is due to the losses of slits and mutual coupling between array elements. The efficiency versus frequency graph is shown in Figure 18. The efficiency of the AVA without corrugations varies from 89.64% to 93.37%, and the efficiency of the AVA with corrugations changes from 91.97% to 94.15%. Thus, the proposed antenna provides good efficiency over the desired operating frequency range.

The importance of the proposed 1 × 4 AVA array was evaluated by comparing it with other recent AVAs, which are given in Table 2. These antennas were compared by focusing on important parameters, such as relative permittivity, dimensions, gain, and frequency bands of antennas. Dimensions are represented in terms of the center frequency of the antenna. The antenna designed in [31,32] provides a very good bandwidth, but its gain variation is higher, and the antenna size is also very large. In [33,34] an antenna is designed with a wide bandwidth and moderate antenna size, but its gain is small. Further, the antennas designed in [1,35,36] are of moderate size and nearly constant gain, but their bandwidth is very small. Moreover, the antenna designed in [37] is compact, but its gain is low. Additionally, in [38] a moderate-sized antenna with a low gain is designed. Recently, a new antenna was designed in 2021 [39], which is of moderate size and moderate gain for 5G applications. The antenna size is smaller in [40] as compared to the proposed antenna, but its gain and bandwidth are lower. Finally, the antenna proposed in [41,42,43] provides a wide bandwidth, but it is considerably large. As compared to these antennas, the proposed antenna is very compact and provides a wide bandwidth and a high and nearly constant gain. Hence, the designed antenna is more appropriate for integration into mm-wave-based 5G communication devices than the above-mentioned antennas.

## 4. Conclusions

A compact 1 × 4 AVA array for 5G mm-wave application is implemented in this paper. The results prove the importance of corrugations in the enhancement of the front-to-back ratio, gain, and bandwidth of antennas. The designed antenna operates in the range of from 24.19 GHz–29.15 GHz to 30.28 GHz–40.5 GHz, which includes three important frequency bands of 5G applications. Moreover, it provides a high and almost constant gain of 8 dBi to 13.2 dBi. Importantly, the improved antenna parameters are achieved without compromising the antenna size, which is 24 mm × 28.8 mm × 0. 254 mm. Furthermore, the radiation patterns of the presented antenna are stable over the operating frequency range. The significance of the proposed antenna is proved by comparing it with the most recent AVAs. This comparison and other antenna results show that the proposed AVA array is suitable for integration in 5G millimeter wave-based communication devices.

## Figures and Tables

**Table 1:** 1 × 4 AVA array dimensions.

Parameters	Dimensions (mm)	Parameters	Dimensions (mm)
$W_{1}$	28.8	$L_{1}$	24
$W_{2}$	5	$L_{2}$	6.3
$W_{3}$	0.4	$L_{3}$	5.4
$W_{4}$	1	$L_{4}$	2
$W_{5}$	0.3	$L_{5}$	2
$W_{6}$	0.2	$L_{6}$	2
$W_{7}$	0.45	$L_{7}$	2
$W_{8}$	0.3	$L_{8}$	2
$W_{9}$	0.3	$L_{9}$	12
$W_{10}$	0.8	$L_{10}$	6.23

**Table 2:** The comparison of the proposed AVA with other AVAs.

Ref. No.	Techniques Employed	$\epsilon_{r}$	Dimensions (${\lambda_{c}}^{3}$)	Gain (dB)	Freq. Band (GHz)
[31]	Fractal	2.94	24.57 × 8.71 × 0.2	0–13.9	4.2–42
[37]	SIW	2.2	2.89 × 1.26 × 0.099	2.15–5.75	11.02–40
[1]	Metamaterial	2.2	7.81 × 3.75 × 0.102	9.2–11.9	24.15–28.5
[36]	DGS	2.2	7.85 × 3.77 × 0.103	9.42–11.44	24.44–28.5
[33]	SIW, DL, Corrugations	2.2	7.4 × 5.27 × 0.076	5–8	58–64
[38]	EBG	4.4	6.796 × 3.398 × 0.063	3–7.25	5–13
[39]	EBG	2.2	9.17 × 5.59 × 0.033	10.4–12.8	26.5–40
[32]	Exponential Strip Lines	2.5	7.37 × 5.84 × 0.047	1–12.5	0.72–17
[41]	Slots	3.38	14.296 × 5.69 × 0.0672	4–8.5	3.2–40
[40]	Kernel Regression	4.4	4.21 × 2.44 × 0.039	−1–6	1–6
[34]	Parasitic Patch	3.5	5.49 × 5.13 × 0.022	2.5–9.8	2.2–12
[35]	Corrugations	2.65	3.24 × 2.52 × 0.036	9–11.5	2.3–11
[42]	Corrugations	3.55	7.91 × 3.165 × 0.061	NG–8	6–18
[43]	Corrugations	3.38	12.16 × 7.22 × 0.03	6.7–15	1.65–18
Proposed AVA Array	Corrugations	2.2	4.6 × 3.83 × 0.04	8–13.2	24.19–29.15, 30.28–40.5

SIW—substrate-integrated waveguide, DGS—defected ground structure, DL—dielectric lens, EBG—electromagnetic bandgap, NA—not applicable, NG—not given

## Data Availability

No database required.
